# Granulovacuolar Degeneration in Brains of Senile Cynomolgus Monkeys

**DOI:** 10.3389/fnagi.2019.00050

**Published:** 2019-03-07

**Authors:** Huda S. Darusman, Dewi Ratih Agungpriyono, Vinka A. Kusumaputri, Dondin Sajuthi, Steven J. Schapiro, Jann Hau

**Affiliations:** ^1^Faculty of Veterinary Medicine, Bogor Agricultural University (IPB), Bogor, Indonesia; ^2^Primate Research Center, IPB, Bogor, Indonesia; ^3^Department of Experimental Medicine, Faculty of Health and Medical Sciences, University of Copenhagen, Copenhagen, Denmark; ^4^Department of Comparative Medicine, The University of Texas MD Anderson Cancer Center, Bastrop, TX, United States

**Keywords:** non-human primate, neurodegenerative disease, Alzheimer’s disease, aging, histopathology, spontaneous lessions

## Abstract

Neurons with histopathological changes consistent with granulovacuolar degeneration (GVD) were found in brain sections from aged cynomolgus monkeys (*Macaca fascicularis*) with clinical and pathological signs of cognitive aging. To our knowledge, this is the first reported description of GVD in non-human primates. GVD-like lesions were found also in age-matched cognitively healthy subjects, albeit in lower numbers, suggesting that they may relate to aging and the increase may have tendency to increase with the memory deficits. The increased incidence of GVD-like lesions in memory-impaired subjects with pahological backgrounds of senile plaques (SPs) and tauopathy is, however, an interesting observation of relevance to the characterization of pathologies in the spontaneous cynomolgus monkey model of human Alzheimer’s type of brain pathology.

## Introduction

Non-human primates (NHPs) with spontaneous pathological lesions similar to those found in human Alzheimer’s disease (AD) are promising models for neurodegenerative studies (Verdier et al., 2015; Perez et al., 2016; Edler et al., 2017). Several tests designed to benchmark memory, developed within the human neuropsychological domain, have now been successfully adapted for use with NHPs. These include delayed response tasks (DRTs), where delays of various durations are imposed between the presentation of a stimulus and a desired response (Rodriguez and Paule, 2009; Nagahara et al., 2010). These types of memory tests are appropriate for assessing cognitive aging in NHPs. Some of the earliest cerebral lesions in the etiology of AD are typically located in the hippocampus (Deiana et al., 2010), making spatial memory tests of particular interest when designing comparative NHP studies to explore cross-species similarities in age-related memory impairments. The connection between hippocampal lesions and spatial memory decline was documented by Rapp (1998) through electrophysiological studies of aged hippocampi, which provided evidence that normal aging significantly influences properties of hippocampal formation thus affecting the status of learning memory. Furthermore, regional specificity of hippocampal circuit-patterns suggested that a decline in the fidelity of input to the hippocampus from the entorhinal cortex plays a critical role in spatial learning (Smith et al., 2000).

Spontaneous formation of senile plaques (SPs) has been studied in several NHP species, including squirrel monkeys (Walker et al., 1987), rhesus monkeys (Cork et al., 1990; Walker, 1997), African green monkeys (Kalinin et al., 2013), cynomolgus monkeys (Nakamura et al., 1998), chimpanzees (Edler et al., 2017) and gorillas (Perez et al., 2016). Our studies of cynomolgus monkeys have identified an age-related decline in DRT performance (Darusman et al., 2013a). We also found that DRT performance was correlated with circulating levels of amyloid-beta (Aβ) levels (Aβ—specifically Aβ_42_; Darusman et al., 2013b; Yue et al., 2014). Expanding our study, we found indications that structural magnetic resonance imaging could be used to identify abnormalities, such as atrophy in the hippocampus and cortical areas, in aged monkeys with poor memory and low circulating Aβ_42_ levels (Darusman et al., 2014b). Immunohistochemical analysis of brain sections from these memory-impaired individuals revealed lesions similar to those associated with human AD: cerebral amyloid angiopathy (CAA), Aβ_42_-positive SPs, and structures reminiscent of neurofibrillary tangles (NFTs) staining positive for phosphorylated tau (Darusman et al., 2014a).

Several other pathological indicators of AD, including neuronal apoptosis, synaptic loss, artifacts in the cytoplasm of hippocampal pyramidal neurons, termed Hirano Bodies (HB), and granulovacuolar degeneration (GVD) have been identified in humans (Serrano-Pozo et al., 2011). GVD is defined as the presence of electron-dense granules within membrane-bound cytoplasmic vacuoles, mainly in hippocampal pyramidal neurons (Okamoto et al., 1991). GVD has also been observed in cognitively healthy elderly people, but is more severe and frequent in age-matched AD patients (Ball, 1978; Xu et al., 1992). In the present investigation, we attempted to identify GVD in sections from brains of aged cynomolgus monkeys suffering from memory impairments consistent with senility (Darusman et al., 2013b, 2014a). We hypothesized that these cynomolgus monkeys, which present with behavioral and physiological changes similar to AD, would also be affected by cerebral GVD.

## Materials and Methods

### Samples

Paraffin-embedded brain sections were collected from six subjects described in Darusman et al. (2013a,b, 2014a). Individuals were aged monkeys (20–25 years old) classified as memory-affected (*n* = 3) or age-matched controls (*n* = 3). Euthanasia was performed according to defined humane endpoints including clinical parameters such as progressive weight loss, paleness of mucous membranes, reduced appetite, and/or general weakness. Subjects were euthanized by pentobarbital and phenytoin injection, followed by saline perfusion and exsanguination; the perfusions were then continued by paraformaldehyde solution (Darusman et al., 2014a). Subjects with memory-deficits and age-matched controls were justified by spatial-memory tests of the DRT performance. The DRT mainly assessed the subjects’ spatial memory of the short-term, long-term and memory-load test (Darusman et al., 2013a).

All procedures involving animals were carried out in accordance with the institution’s approved standard operating procedure which are based on The Guide for the Care and Use of Laboratory Animals by NRC 2011. The subjects’ housing conditions and test procedures were approved by the Institutional Animal Care and Use Committee (ACUC) of The Primate Research Center (PRC), Bogor Agricultural University (ACUC No. IPB PRC-13-A002).

### Histopathology and Scoring

The paraffin-embedded brain sections of the six subjects were trimmed into 5-μm sections with a microtome, stained with hematoxylin-eosin (HE) and mounted with *entellan*. The tissue sections were studied by light microscopy (40× and 100× magnifications) and scored by two pathologists—one at the Faculty of Veterinary Medicine, Bogor Agricultural University (IPB) and the other at the Laboratory of Pathology, Primate Research Center, IPB. Both pathologists worked independently and were blinded to the identity of the samples. GVD lesions were identified based on morphology, size, granules and vacuoles, and HE uptake. For the quantitative analysis, the scoring of Gibson-Corley et al. (2013) was employed. The number of lesions were counted in a subset of 100 neurons from the hippocampus and the mediotemporal lobe. The lesions were categorized as GVD if they met the following criteria.

1. Cells are enlarged and often not shaped in their basic form (triangular/pyramidal; Zaidel et al., 1997).
2. Clear space in the cytoplasm—translucent vacuoles that fail to stain in cytoplasm (Myers and Mc Gavin, 2007).
3. Large (up to 5 μm) membrane-bound vacuoles (Funk et al., 2011).
4. Presence of basophilic granules within the vacuoles contain fragments of cell components (e.g., mitochondria, endoplasmic reticulum) destined for destruction (Cotran et al., 1999).
5. Granules are coarse and/or morphologically shaped as a vesicle with a surrounding double unit membrane (Funk et al., 2011).

## Results

**Figure 1:**
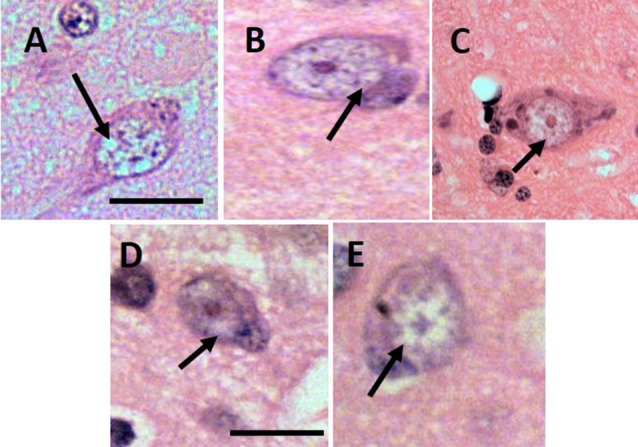
Lesion of granulovacuolar degeneration (GVD) from 9661 **(A)**, 10749 **(B)**, I1112 **(C)**, T3311 **(D)**, 10063 **(E)**. Arrows indicate the suspected GVD lesion. Scale bars: 20 μm.

Based on human’s GVD criteria and by comparing our histopathology samples with images of GVD in AD patients (Yamazaki et al., 2011), the lesion indicative of GVD were found in all aged subjects (Figure 1). The size of the vacuoles was calculated as minimum 7–8 μm and the largest was 15–17 μm. Morphologically the cells were shaped as rounded and not in their basic triangular/pyramidal forms. The vacuoles were similar to human GVD with clearance of cytoplasm space, translucent and with basophilic-coarsed granules. The number of GVD lesions was greater in the senile subjects compared to age-matched control subjects (Table 1), with exception to subject 10063 that had the lowest GVD score among senile subjects. Based on our imaging study (Darusman et al., 2014b), cerebral atrophy and hippocampal atrophy were morphologically diagnosed in memory-affected subjects compared with the age-matched subjects. Our findings seems to agree with humans GVD becuase the lesions were uniformly found in aged suspects and tended to be more frequent in individuals with spatial memory deficit (Köhler, 2016).

**Table 1:** Quantitative analysis of the lesions with subject characteristics.

Subject (ID/sex/classification)	Total DRT (%)	Lesions and affected lobes or area			Neurons with GVD-like lesions (per 100 neurons)		
		SP	CAA	Tau	Mediotemporal	Hippocampal	Total
9661/male/control	60	-	F, T, P	-	3	4	7
10749/female/control	62	-	F, T, H	-	5	0	5
T3283/male/control	66	-	F, T, O	-	0	0	0
T3311/male/memory-affected	40	F, P, H	F, P, O	-	5	7	12
10063/female/memory-affected	41	-	F, T	-	3	0	3
I1112/female/memory-affected	34	F, T, O, P, H	F, T, O, P, H	F, T, O	17	0	17

*Data on delayed response test (DRT) performance are from Darusman et al. (2013a,b). Data on lesions are from Darusman et al. (2014a). Total DRT performance is presented as the percent of trials where subjects could correctly retrieve hidden item(s) in a set of memory tests. The lobes of the brain harboring lesions are encoded as F (frontal), T (Temporal), O (occipital), and P (parietal) and area of hippocampal (H)*.

## Discussion

The lesions indicative of GVD in the brains of aged cynomolgus monkeys appear similar to human GVD with respect to size, shape, and morphology of the granules and vacuoles (Funk et al., 2011; Yamazaki et al., 2011). Vacuolar degeneration, or hydropic degeneration, is a commonly used term to describe the microscopic appearance of acute cell swellings that occur in neurons and glial cells of the brain, endothelium, epithelium, alveolar pneumocytes, hepatocytes, and renal tubular epithelial cells (Myers and Mc Gavin, 2007). The present findings may identify an additional histopathological marker linking human AD and age-associated cognitive impairments in cynomolgus monkeys. All subjects shared the pathology of CAA in the brain, with only memory-affected subjects additionally having the SPs in hippocampus and indication of Tauopathy (Darusman et al., 2014a). Based on the previous study (Darusman et al., 2014a), among the memory-affected subjects, subject 10063 had the lowest pathology score of amyloid disorders and only categorized as CAA, while the other memory-affected individuals shared SPs and CAA. It is perhaps not surprising that the less-severe pathological lesions in 10063 are associated with a lower score in GVD as well, considering that the vacuoles of GVD contain protein related with tau pathology, autophagy, diverse signal-transduction pathways, cell stress and apoptosis (Köhler, 2016).

Differences in localization of both amyloid plaques and fibrillary tangles in cynomolgus monkeys suggest that the pathogenesis of Alzheimer’s-like proteopathies in NHPs may be subtly different from human AD (Oikawa et al., 2010; Heuer et al., 2012; Darusman et al., 2014a); in the human disease, both lesions are found in the same regions of the brain. These findings suggest that AD is a uniquely human condition with NHP models instead describing brain aging and Aβ-amyloidosis in the absence of NFT (Heuer et al., 2012; Perez et al., 2016; Edler et al., 2017). The suspected GVD identified in the present study supports the existence of other pathological hallmarks of AD in NHPs, and possibly other large animals (Schmidt et al., 2015; Hainsworth et al., 2017) that may be relevant for comparative studies and for the use of the cynomolgus monkey as a spontaneous model of human neurodegenerative disease of Alzheimer’s type.

GVD-like lesions were found in individuals of both of our studied groups, suggesting that GVD may be related to normal aging in cynomolgus monkeys. The higher incidence of neurons with GVD-like lesions in our memory-affected subjects is, however, an interesting observation that merits more investigation. Earlier studies of neurons in the hippocampus field demonstrate that the degeneration among principal neurons in the hippocampus is not responsible for behavioral impairments in aged individuals and may be associated with normal aging as well as the final common pathway mediating age-related functional decline (Rapp and Gallagher, 1996).

In humans, GVD has been described as a pathological hallmark of AD outside the SPs and NFT. The frequency of GVD correlates with amyloid plaques and NFT (Yamazaki et al., 2011), and an increase in GVD parallels the severity of AD symptomology (Ball and Lo, 1977), specifically the decline of episodic memory performance (Ghoshal et al., 1977). Further studies of GVD composition and origin have offered additional insights (Funk et al., 2011). Immunohistochemistry has revealed a possible link between GVD and AD-related neurodegeneration. Caspase 3, an apoptotic effector protease involved in cleavage of tau (Gamblin et al., 2003) and amyloid precursor protein (APP; Gervais et al., 1999), a causative factor generating deposition of amyloid fibrils into SP (Blennow et al., 2010; Hampel et al., 2010; Frisoni, 2012) have been found in GVD, but rarely in other pathological structures (Jellinger and Stadelmann, 2000; Su et al., 2002). APP is ubiquitously expressed in neurons of macaque species, including rhesus, cynomolgus, and lion-tailed macaque, and apes including chimpanzee and gorilla (Martin et al., 1991; Heuer et al., 2012; Perez et al., 2016; Edler et al., 2017).

In the present study, the suspected GVD was discovered principally in the mediotemporal lobes, which agrees with the tau pathology described by Oikawa et al. (2010) and Heuer et al. (2012). The individual with the most extensive GVD was a memory-affected subject who also presented with the highest levels of circulating SP, CAA, and Tau (subject I1112).

In conclusion, GVD-like lesions were found in all aged cynomolgus monkeys studied. Their occurrence in cognitively normal aged subjects suggests that the lesions are associated with aging. Their increased frequency in memory-affected subjects indicates an association with, or involvement in, spatial working memory deficits. The GVD identified in cynomolgus monkeys in the present study seems similar to human GVD with regard to pathology and with an increase in prevalence in subjects with spatial memory deficits thus correlated with the higher number of affected neurons in the hippocampus area.

## Author Contributions

HD has contributed in designing and writing most of the manuscript and submitting the manuscript for publication. DA has contributed in assuring the methods and the quality of the results. VK with HD and DA have determined the criteria for histopathology analysis, and conducted most of the laboratory works. DS shared his role in revising important intellectual matters related to degenerative studies involving NHPs as animal models. SS validated the study design, particularly in terms of the behavioral and memory components of the work. JH contributed with writing and reviewing the manuscript and approving for publication of the content.

## Conflict of Interest Statement

The authors declare that the research was conducted in the absence of any commercial or financial relationships that could be construed as a potential conflict of interest.
